# Reproducibility study on myocardial strain assessment using fast-SENC cardiac magnetic resonance imaging

**DOI:** 10.1038/s41598-018-32226-3

**Published:** 2018-09-20

**Authors:** Sorin Giusca, Grigorios Korosoglou, Victoria Zieschang, Lukas Stoiber, Bernhard Schnackenburg, Christian Stehning, Rolf Gebker, Burkert Pieske, Andreas Schuster, Sören Backhaus, Elisabeth Pieske-Kraigher, Amit Patel, Keigo Kawaji, Henning Steen, Tomas Lapinskas, Sebastian Kelle

**Affiliations:** 1 Department of Cardiology and Vascular Medicine, GRN Hospital Weinheim, Weinheim, Germany; 2 Department of Internal Medicine/Cardiology, German Heart Center Berlin, Berlin, Germany; 3 Philips Healthcare, Hamburg, Germany; 4 Department of Cardiology, Medical Academy, Lithuanian University of Health Sciences, Kaunas, Lithuania; 5 Department of Cardiology and Pneumology, University Medical Center, Georg-August University, Göttingen, Germany; 6 Department of Internal Medicine/Cardiology, Charité Campus Virchow Clinic, Berlin, Germany; 7 Department of Medicine, University of Chicago, Chicago, Illinois USA; 8 Department of Biomedical Engineering, Illinois Institute of Technology, Chicago, Illinois USA; 9 Department of Internal Medicine/Cardiology, Marienkrankenhaus Hamburg, Hamburg, Germany; 10 DZHK (German Centre for Cardiovascular Research), Partner Site, Berlin, Germany; 11 Department of Cardiology, Royal North Shore Hospital, the Kolling Institute, Northern Clinical School, University of Sydney, Sydney, Australia

## Abstract

Myocardial strain is a well validated parameter for estimating left ventricular (LV) performance. The aim of our study was to evaluate the inter-study as well as intra- and interobserver reproducibility of fast-SENC derived myocardial strain. Eighteen subjects (11 healthy individuals and 7 patients with heart failure) underwent a cardiac MRI examination including fast-SENC acquisition for evaluating left ventricular global longitudinal (GLS) and circumferential strain (GCS) as well as left ventricular ejection fraction (LVEF). The examination was repeated after 63 [range 49‒87] days and analyzed by two experienced observers. Ten datasets were repeatedly assessed after 1 month by the same observer to test intraobserver variability. The reproducibility was measured using the intraclass correlation coefficient (ICC) and Bland-Altman analysis. Patients with heart failure demonstrated reduced GLS and GCS compared to healthy controls (−15.7 ± 3.7 vs. −20.1 ± 1.4; p = 0.002 for GLS and −15.3 ± 3.7 vs. −21.4 ± 1.1; p = 0.001 for GCS). The test-retest analysis showed excellent ICC for LVEF (0.92), GLS (0.94) and GCS (0.95). GLS exhibited excellent ICC (0.99) in both intra- and interobserver variability analysis with very narrow limits of agreement (−0.6 to 0.5 for intraobserver and −1.3 to 0.96 for interobserver agreement). Similarly, GCS showed excellent ICC (0.99) in both variability analyses with narrow limits of agreement (−1.1 to 1.2 for intraobserver and −1.7 to 1.3 for interobserver agreement), whereas LVEF showed larger limits of agreement (−14.4 to 10.1). The analysis of fast-SENC derived myocardial strain using cardiac MRI provides a highly reproducible method for assessing LV functional performance.

## Introduction

Quantitative assessment of left ventricular (LV) functional performance remains one of the cornerstones in the diagnosis and treatment of cardiovascular patients. LV ejection fraction (LVEF) is the most used clinical parameter in evaluating left ventricular performance. Many clinical decisions in patients with cardiovascular conditions are based on the absolute value of LVEF^1^. However, several studies have pointed out the dependence of this parameter on the loading conditions of the heart at the time of the measurement^2^. In addition, in a variety of cardiovascular diseases, LVEF is not able to detect subtle changes in myocardial performance, usually seen in the early phases of the disease^3^. Thus, parameters of myocardial deformation such as myocardial strain were developed with the purpose of better characterization of myocardial regional function. Numerous studies have shown the utility of myocardial strain in the diagnosis of several pathologies, identifying subclinical myocardial changes as well as providing strong prognostic value for future cardiac events^4,5^.

Several non-invasive methods can be employed for extracting myocardial strain. Echocardiography, using either tissue Doppler imaging or speckle tracking, provides a readily available technique for measuring myocardial strain^6,7^. Numerous studies showed the incremental value of echocardiographic strain in identifying subclinical dysfunction in cancer patients and establishing prognosis in patients with ischemic heart disease^8^. However, dependency on image quality, high inter- and intraobserver reproducibility as well as inter-vendor variability hampered the widespread use of this technique in the clinical routine. Cardiac magnetic resonance (CMR) imaging, on the other hand, offers an integrative approach in the evaluation of the LV, providing information regarding morphology, function, hemodynamics and tissue characterization, all in one examination and without exposing the patient to ionizing radiation. Additionally, regional myocardial function can be measured using CMR. One of the first techniques employed in extracting myocardial strain was myocardial tagging^9^. The method was shown to have a good reproducibility, with low coefficient of variations for interobserver and intraobserver analysis^10^. Although it proved useful in identifying viable myocardium and evaluating diastolic function, the method is still hampered by low spatial resolution, long acquisition times and even longer post-processing times^11^. Phase velocity mapping (PVM), another method employed in the early days of CMR for extracting myocardial deformation parameters, uses the same principles that apply for extracting flow velocities. However, the need for measuring relatively low myocardial velocities compared to blood flow velocities results in longer acquisition times and phase distortion^12^. Displacement encoded with simulated echoes (DENSE) is a more recent method developed for measuring myocardial mechanics^13^. *In vitro* studies showed a very good accuracy in measuring deformation^14^. Furthermore, the method provided reproducible results in animal and human studies^15,16^. Although the technique involves fast post-processing, the technical aspects needed for measuring displacement may result in a low signal to noise ratio^12^. In contrast, fast strain encoded CMR imaging (fast-SENC) provides real-time acquisition of myocardial strain in a single heart beat^17^. The method is well validated *in vitro* and was shown to be clinically relevant in identifying myocardial ischemia in low dose dobutamine stress examinations^18^.

The aim of our study was to evaluate the reproducibility at interstudy, interobserver and intraobserver levels for measuring longitudinal and circumferential strain using fast-SENC technique in a population of healthy volunteers and patients with heart failure (HF).

## Methods

Eighteen individuals (11 healthy volunteers and 7 patients with HF – 4 patients with HF with preserved LVEF and 3 patients with HF with reduced LVEF) underwent a CMR scan including fast-SENC acquisitions. The study was approved by the Ethics Committee of the Charité-University Medicine Berlin and complied with the Declaration of Helsinki. All individuals gave written informed consent before participating in the study.

### Cardiac magnetic resonance

The acquisition was performed on a 1.5 Tesla MRI scanner (Achieva, Philips Healthcare, Best, The Netherlands) using a five-element phased array cardiac coil for signal reception. Data acquisition was triggered on the R-wave using a 4-lead vector ECG.

### Fast-SENC acquisition

A previously described^17^ SENC method based on the acquisition of two images with different frequency modulation was employed. Bright regions in the two frequency modulation images represent static and contracting tissues, respectively. Circumferential and longitudinal strains in a range from 5% to −30% were encoded, where negative values represent positive contraction. A real time SENC variant with single-shot spiral readouts was employed. Typical SENC imaging parameters were as follows: field-of-view = 256 × 256 mm², slice thickness 10 mm, voxel size 4 × 4 × 10 mm³, reconstructed resolution at 1 × 1 × 10 mm^3^ using zero-filled interpolation (in-plane ZIP 1024), single-shot spiral readout (3 interleaves) with acquisition time TA = 10 ms, flip angle = 30°, effective echo time (TE) = 0.7 ms, repetition time (TR) = 12 ms, temporal resolution = 36 ms, typical number of acquired heart phases = 22, spectrally selective fat suppression (SPIR), total acquisition time per slice <1 s. Data were acquired in three long-axis (four-, three- and two-chamber) views, and three short-axis views at different LV levels (basal, mid-ventricular and apical).

### Image analysis

All fast-SENC images were uploaded from the scanner into dedicated MyoStrain software (Myocardial Solutions, Inc., Morrisville, North Carolina, USA). All images were analyzed by one observer (SG). Before starting the analysis, the observer was trained by a representative of the software company with an emphasis on possible sources of error. The LV circumferential strain was extracted from three long-axis views, whereas longitudinal strain was extracted from three short-axis images (basal, mid-ventricular and apical). The endocardial and epicardial borders were drawn at the end-systolic cardiac phase and after application of the automatic tissue tracking algorithm software, the borders were traced throughout the entire cardiac cycle. The tracking was verified and manually corrected when needed for each segment. In addition, LVEF was calculated from all three long-axis images. A 16-segment model was used for the longitudinal strain and an 18-segment model for the circumferential strain. Global longitudinal strain (GLS) was expressed as the average value of all 16 segments obtained from the short-axis views. Similarly, global circumferential strain (GCS) was the average value of the 18 segments obtained in the long-axis views. As myocardial shortening occurs in both longitudinal and circumferential directions in systole, the strain values are consequently negative and are so reported. However, throughout the text, and in keeping with most of the literature on the subject, we will refer to the absolute values (i.e. higher strain values meaning more deformation and consequently more “negative” values).

### Interstudy variability

**Figure 1:**
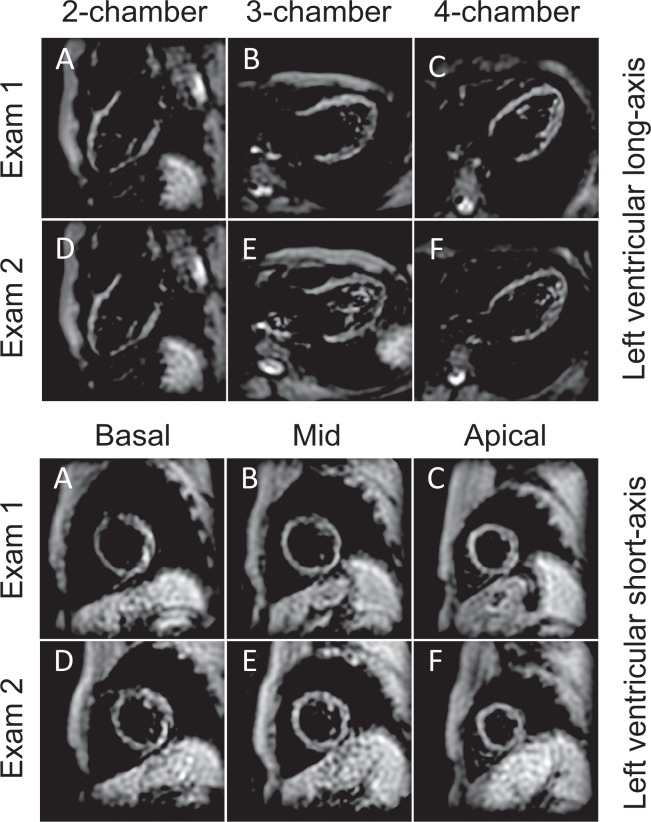
The upper two rows demonstrate fast-SENC images acquired in LV two- (**A**,**D**), three- (**B**,**E**) and four-chamber (**C**,**F**) views of a healthy volunteer during baseline and follow-up CMR studies. Acquisition was performed at the same level of the heart. Lower two rows images demonstrate three short-axis views at LV basal (**G**,**J**), mid-ventricular (**H**,**K**) and apical (**I**,**L**) level. LV = left ventricular; CMR = cardiac magnetic resonance.

**Figure 2:**
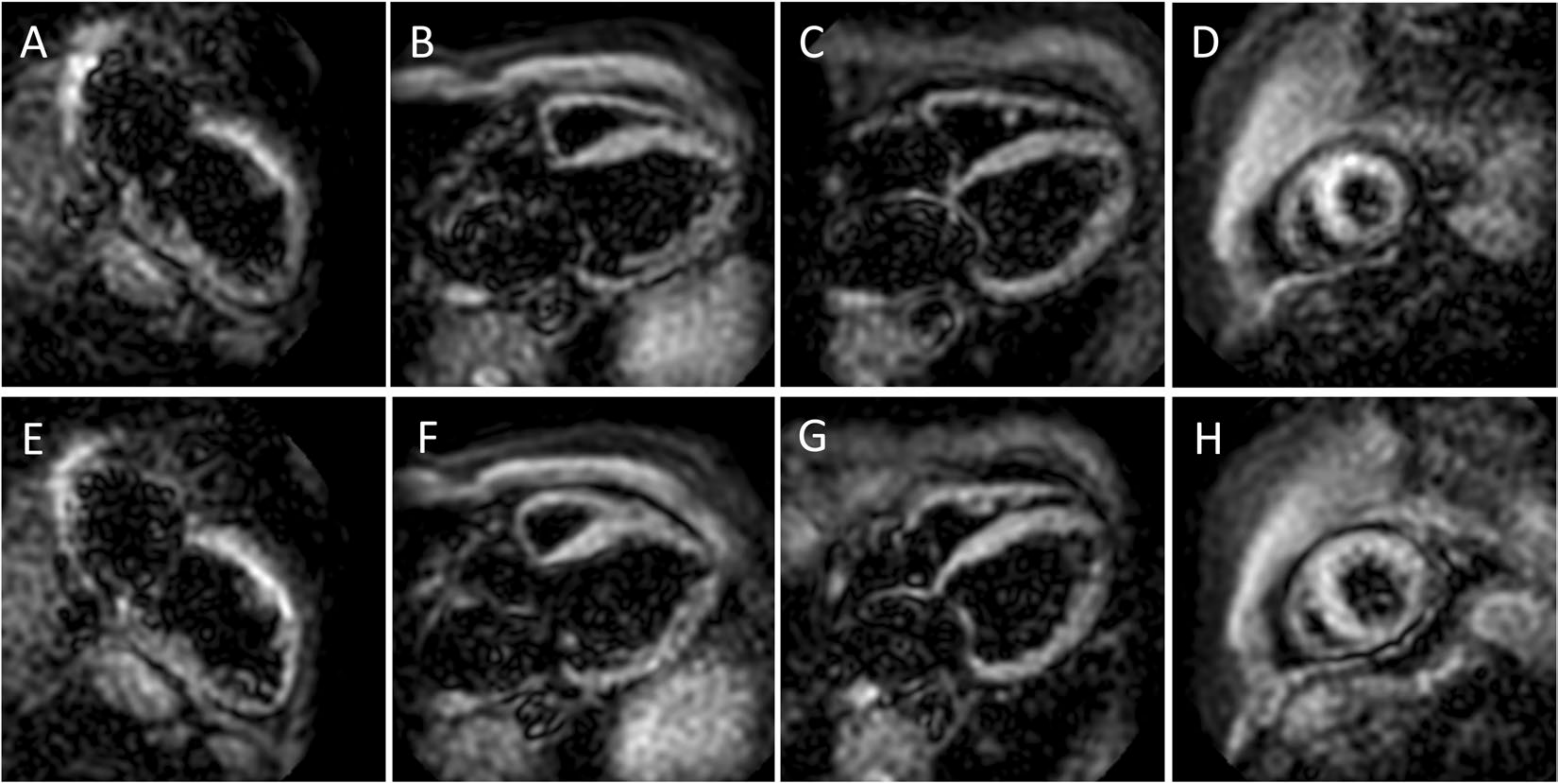
Demonstrating identical CMR images as in Fig. 1 acquired in a patient with heart failure with reduced LV ejection fraction (HFrEF). CMR = cardiac magnetic resonance; LV = left ventricular.

All 18 individuals underwent a second identical CMR examination at a median of 63 [range 49‒87] days after the first examination. To avoid recollection bias of the involved CMR staff, a minimum of 6 weeks difference have been between the first and second scan. Care was taken that acquisitions were performed at the same levels of the heart (Figs 1 and 2) and that in heart failure patients no change in symptoms and medication occurred. In addition, in healthy subjects new onset of cardiac disease was excluded.

### Intra- and interobserver reproducibility

To test for intraobserver variability, ten random datasets were reanalyzed 1 month after the first analysis by the first observer (SG), blinded to the results of the first analysis. For interobserver variability these 10 datasets were analyzed by a second observer (VZ) who was blinded to the results of the first observer.

### Statistical analysis

Data are expressed as mean ± standard deviation. A t-test was used to compare continuous variables. Test-retest, interobserver and intraobserver variability were tested using intraclass correlation coefficient (ICC) and Bland-Altman analysis. The following levels of agreement were used: excellent for ICC >0.74, good for ICC 0.6‒0.74, fair for ICC 0.4‒0.59, poor for ICC <0.4^19^. In addition, for the test-retest variability, the coefficient of variation was calculated. Receiver operator analysis (ROC) analysis was used to determine the optimal value for GLS and GCS, respectively, to identify a reduced LVEF (LVEF <50%). A p value <0.05 was considered statistically significant. All data was analyzed using MedCalc Statistical Software (Version 12.7.2, Ostend, Belgium).

## Results

**Figure 3:**
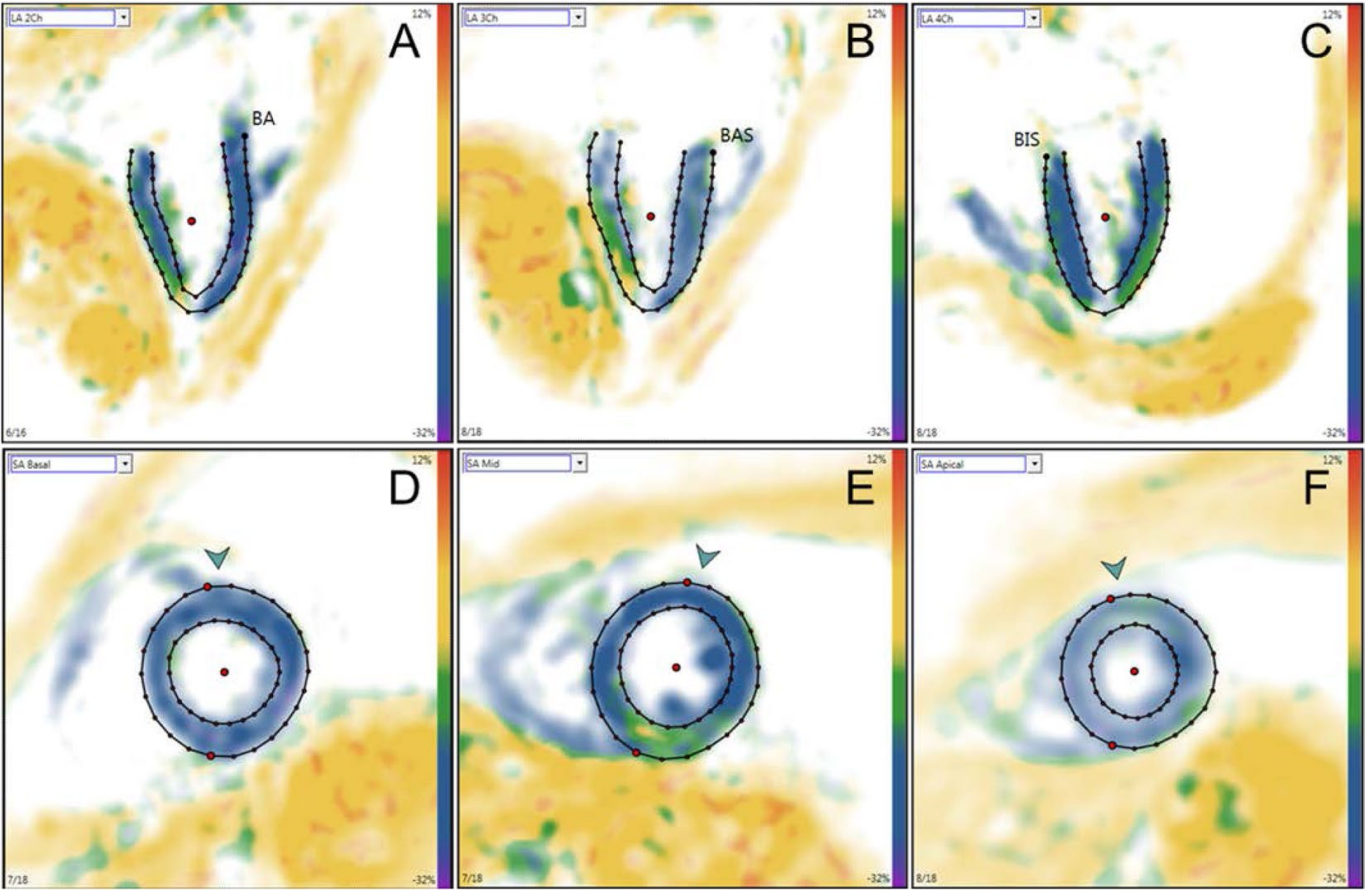
The images of the upper row demonstrate LV two- (**A**), three- (**B**) and four-chamber (**C**) views at end-systolic phase used to calculate LV volumes, mass-, and ejection fraction as well as LV global and segmental circumferential strain. The lower row images show short-axis views at LV basal (**D**), mid-ventricular (**E**) and apical (**F**) level used to derive global and segmental LV longitudinal strain. LV = left ventricular.

Table 1 represents the demographic and standard CMR data of the studied population. Patients with HF demonstrated more dilated LV and significantly reduced LVEF than healthy individuals. Moreover, the strain values (GLS and GCS) were significantly lower (in absolute value) than in healthy controls. A typical strain analysis in a healthy individual can be appreciated in Fig. 3.

**Table 1:** Demographic and functional parameters of study population.

	Healthy individuals (n = 11)	Heart failure patients (n = 7)	P value
Age (years)	28.2 ± 4.81	73.3 ± 8.64	<0.001
Male gender	6 (54.5)	4 (57.1)	0.92
Heart rate (bpm)	69.2 ± 10.78	61.3 ± 8.01	0.14
LVEDV (ml)	164.8 ± 24.7	200.0 ± 56.2	0.08
LVEDVi (ml/m^2^)	89.3 ± 10.7	101.1 ± 21.3	0.13
LVESV (ml)	64.8 ± 18.8	138.6 ± 61.6	0.001
LVESVi (ml/m^2^)	34.7 ± 7.6	69.6 ± 26.6	<0.001
LVEF (%)	61.3 ± 6.3	33.0 ± 14.8	<0.001
LVEDM (g)	95.4 ± 21.1	130.6 ± 40.9	0.02
LVEDMi (g/m^2^)	51.4 ± 7.8	66.0 ± 15.6	0.01
GLS (%)	−20.1 ± 1.4	−15.7 ± 3.7	0.002
GCS (%)	−21.4 ± 1.1	−15.3 ± 3.7	0.001

Results are reported as mean ± standard deviation or total number (percentage). LVEDV = left ventricular end-diastolic volume; LVEDVi = left ventricular end-diastolic volume index; LVESV = left ventricular end-systolic volume; LVESVi = left ventricular end-systolic volume index; LVEF = left ventricular ejection fraction; LVEDM = left ventricular end-diastolic mass; LVEDMi = left ventricular end-diastolic mass index; GLS = global longitudinal strain; GCS = global circumferential strain. CMR-data are derived from the first examination.

### Interstudy variability

**Figure 4:**
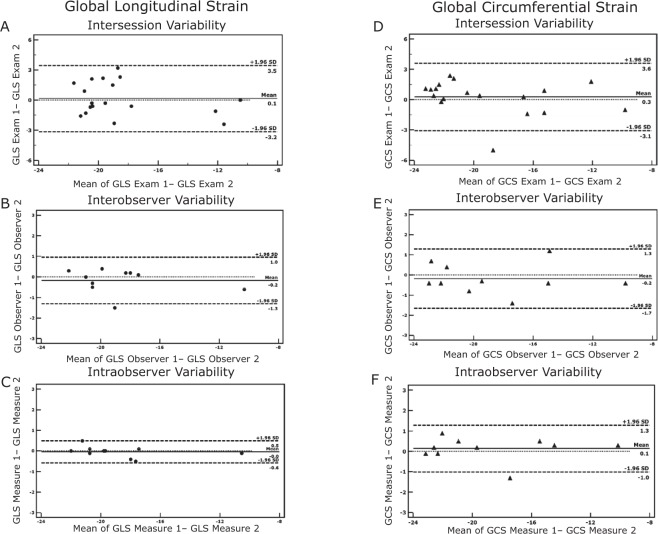
Bland-Altman plots with limits of agreement (1.96 SD) for the intersession (**A**), interobserver (**B**) and intraobserver (**C**) variability as shown in the measurements of LV global longitudinal strain. Similar plots with limits of agreement (1.96 SD) for the intersession (**D**), interobserver (**F**) and intraobserver (**G**) variability as shown in the measurements of LV global circumferential strain. LV = left ventricular.

We found excellent intersession reproducibility of measurements of GLS and GCS as shown by the respective ICC (0.93 and 0.95 for GLS and GCS respectively). In addition, the Bland-Altman analysis showed narrow limits of agreement for these two parameters (Table 2). Similarly, the two measurements of LVEF exhibited an excellent ICC (0.96). However, the limits of agreement between the two examinations were larger than those seen for strain measurements. The Bland-Altman analysis is shown in Fig. 4.

**Table 2:** Interstudy reproducibility for LV ejection fraction and global myocardial strain parameters.

Parameter	Exam 1	Exam 2	Mean difference	Limits of agreement	ICC (95% CI)	CoV
LVEF	50.3 ± 17.4	52.4 ± 15.1	−2.1	−14.4 to 10.1	0.92 (0.80 to 0.97)	8.8%
GLS	−18.4 ± 3.3	−18.6 ± 3.7	0.1	−3.2 to 3.5	0.94 (0.84 to 0.98)	−6.2%
GCS	−19.0 ± 3.9	−19.3 ± 4.4	0.3	−3.1 to 3.6	0.95 (0.88 to 0.98)	−6.1%

Results are reported as mean ± standard deviation. LVEF = left ventricular ejection fraction; GLS = global longitudinal strain; GCS = global circumferential strain; ICC = intraclass correlation coefficient; CI = confidence interval; CoV = coefficient of variance.

### Inter and intraobserver reproducibility

In both inter and intraobserver analysis, the reproducibility of strain measurements and LVEF was excellent (Tables 3 and 4). Moreover, both GLS and GCS showed very narrow limits of agreement with LVEF again showing larger limits of agreement.

**Table 3:** Interobserver reproducibility for LVEF and myocardial strain.

Parameter	First observer	Second observer	Mean difference	Limits of agreement	ICC (95% CI)
LVEF	49.7 ± 16.3	49.2 ± 14.9	0.5	−11.0 to 12.0	0.96 (0.85 to 0.99)
GLS	−18.8 ± 3.2	−18.6 ± 3.4	−0.1	−1.3 to 0.96	0.99 (0.96 to 0.99)
GCS	−18.8 ± 4.3	−18.6 ± 4.4	−0.2	−1.7 to 1.3	0.99 (0.96 to 0.99)

Results are reported as mean ± standard deviation. All abbreviations as in Table 2.

**Table 4:** Intraobserver reproducibility for LVEF and myocardial strain.

Parameter	First measurement	Second measurement	Mean difference	Limits of agreement	ICC (95% CI)
LVEF	49.7 ± 16.3	49.7 ± 17.6	0.1	−9.8 to 9.9	0.97 (0.90 to 0.99)
GLS	−18.8 ± 3.2	−18.8 ± 3.3	−0.04	−0.6 to 0.5	0.99 (0.97 to 0.99)
GCS	−18.8 ± 4.3	−18.9 ± 4.3	−0.2	−1.0 to 1.2	0.99 (0.97 to 0.99)

Results are reported as mean ± standard deviation. All abbreviations as in Table 2.

### ROC Analysis

**Figure 5:**
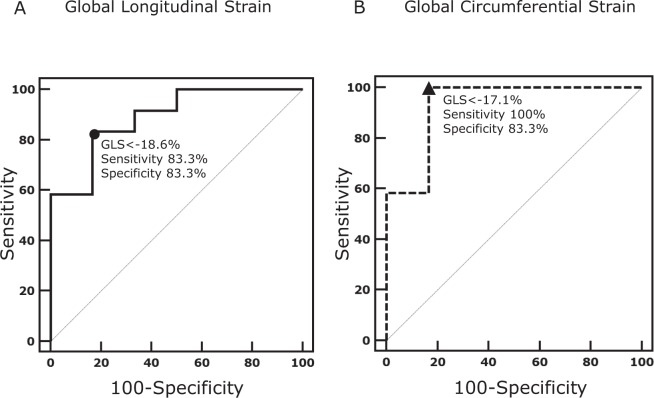
ROC analysis for determining the optimal value for global longitudinal strain (**A**) and global circumferential strain (**B**) in identifying patients with a reduced LV ejection fraction. ROC = received operating characteristic; LV = left ventricular.

A value of −18.6% for GLS identified a LVEF <50% with sensitivity of 83.3% and specificity of 83.3%. Similarly, a GCS value of −17.1% identified a LVEF <50% with 100% sensitivity and 83.3% specificity (Fig. 5).

## Discussion

We evaluated 18 healthy individuals and patients with heart failure using fast-SENC for measuring GLS, GCS and LVEF. We found (i) excellent interstudy as well as interobserver and intraobserver agreement for these functional LV parameters. (ii) Fast-SENC derived GLS and GCS allows very good discrimination between patients with normal LV performance and those with reduced LVEF.

Several CMR techniques can be employed for extracting myocardial deformation parameters. Historically, myocardial tagging was the first technique used to measure myocardial strain and it later became the standard to which other techniques were compared. Although myocardial tagging is well validated through several *in vitro* studies, the widespread use of this method is still hampered by long acquisition and post-processing times^20,21^. SENC was first proposed in 2001 as a method to extract longitudinal strain from short-axis images using harmonic phase MRI^22^. For the strain values to be extracted, out of plane phase-encoding gradients orthogonal to the image plane are employed. Thus, short-axis images (basal, mid-ventricular and apical) are used to generate longitudinal strain and long-axis images (four-chamber, two-chamber and three-chamber) to obtain circumferential strain. At first, the method was difficult to implement because of long breath-hold periods necessary for the acquisition of multiple heart beats. The method improved, ultimately allowing image acquisition in a single heartbeat – fast-SENC^17^. The fast-SENC method was validated *in vitro* as well as in normal human subjects. Strain values derived using fast-SENC protocols correlated closely to values derived from CMR-tagging^23^. In addition, the method proved superior to visual wall motion analysis during dobutamine stress CMR and allowed the diagnosis of coronary artery disease at lower stages of stress^24^. Another advantage of the method is its ability to provide information related to diastolic function of the LV^25^. Thus, it enabled better assessment of the diastolic dysfunction of patients with diabetes mellitus. The method was shown to be useful in the analysis of the right ventricle, offering better characterization of right ventricular performance in healthy individuals and patients with pulmonary hypertension^26^.

In our study, we analyzed strain values on a wide range of LVEF. We found that both GLS and GCS have very good discriminatory power in differentiating between normal and reduced LVEF.

An abundance of data exists confirming the role of myocardial strain, derived using either echocardiography or CMR, in the diagnosis, treatment and prognosis of various cardiovascular conditions. However, a lack of standardization still hampers the implementation of parameters of myocardial deformation imaging in clinical practice. This is mainly due to still unsatisfactory high variability in measurements. High inter- and intraobserver and test-retest variability reduces the consistency of the method. This in turn makes reliable detection of small changes in regional myocardial function difficult to achieve. A recent study by Amzulescu *et al*. compared strain values extracted with echocardiography and CMR, and found a very large variation between the two methods as well as a significant bias in the echocardiographic derived strain measurements^27^. Consequently, in the light of their findings, the authors questioned the clinical usefulness of echo-derived strain measurements. In addition, strain measurements obtained with echocardiography are vendor dependent, as shown by a recent study by Mirea *et al*.^28^. The authors found high variability between different vendors in strain values. The ICC between different vendors was as low as 0.52. In addition, as much as 22% of segments had to be excluded from the analysis due to low image quality. SENC acquisition, on the other hand, is vendor independent and can be implemented in the MRI machines of different vendors.

The data from studies performed on CMR derived myocardial strain show better reproducibility compared to echocardiography. An initial study performed on 24 participants showed very good agreement in the measurement of circumferential strain using myocardial tagging^29^. Two more recent studies confirmed the good reproducibility of myocardial strain derived with tagging techniques^10,30^. However, in the study by Donekel *et al*., the ICC for interstudy reproducibility was lower for the same parameters^30^. A validation study by Kar *et al*. showed very narrow limits of agreement between repeated measurements for circumferential strain extracted with DENSE^16^. A study by Miyagi *et al*. performed in 24 patients with suspected coronary artery disease noted an excellent interobserver agreement with an ICC >0.95 for estimating myocardial strain^31^. Similar results were found in a more recent study performed in 17 healthy volunteers^32^. Similar to myocardial tagging, however, the agreement was moderate when testing for interstudy variability.

In recent years, feature tracking (FT) has been used for deriving deformation parameters from conventional cine CMR acquisitions. The method tracks individual “features” of a predetermined region of interest throughout the entire cardiac cycle. The main advantage of the method is that no additional acquisition is necessary during the CMR scan. When compared to CMR-tagging, the consistency of FT was not uniform between studies^33,34^. In addition, the two studies with the highest number of healthy controls do not have overlapping values for longitudinal and circumferential strain^35,36^. As seen with echocardiography, different software from different vendors produces inconsistent results as shown by Schuster *et al*.^37^. Lastly, the limits of agreement when testing the reproducibility of strain measurements using FT are less narrow compared to those in our study^38^.

To the best of our knowledge, this the first study to test the reproducibility (including interstudy variability) of strain measurements derived with fast-SENC. In comparison to other methods, we showed excellent ICC with very narrow limits of agreement for both longitudinal and circumferential strain. Thus, fast-SENC derived strain measurements are suitable for serial studies and could reliably detect subclinical changes in myocardial function.

### Limitations

The main limitation of our study is related to small population size. However, this is in concordance with most of the reproducibility studies to date. In addition, our sample size included both healthy individuals and patients with reduced LVEF.

## Conclusion

The analysis of fast-SENC derived myocardial strain using cardiac MRI provides a highly reproducible method for assessing left ventricular performance.
